# Experimental Investigation of Damage Formation in Planar Fibrous Networks During Stretching

**DOI:** 10.1038/s41598-019-39460-3

**Published:** 2019-02-26

**Authors:** Evelin Sipos, Tatsuo Kaneko, Miklós Zrinyi

**Affiliations:** 1 Laboratory of Nanochemistry, Department of Biophysics and Radiation Biology, Semmelweis University, H-1089 Nagyvárad tér 4, Hungary; 2 Japan Advanced Institute of Science and Technology, Energy and Environmental Area, 1-1 Asahidai, Nomi, Ishikawa, 933-1212 Japan

## Abstract

This paper presents the results of unidirectional strain-controlled experiments on fibrous electrospun networks used to study damage formation during elongation. The experimental loading curve shows a symmetrical parabolic type dependence at large scale and saw tooth-like force−extension behaviour at small scale. The damage formation was quantified by determining the number and the magnitude of abrupt force drops. The experiments evidenced that damage evolution is a consequence of strain induced random events. The frequency distribution of the number of damages as well as the magnitude of rupture force were represented by histograms. The results of the present study provide a better insight into damage tolerance and complex nonlinear tensile properties of electrospun networks. In addition, it could suggest a possible probabilistic approach to the fiber bundle model which has mainly motivated this study.

## Introduction

Fibrous materials have important technological applications owing to their excellent mechanical performance and low weight. The mechanical properties of fibre assemblies depends on the strength and the toughness of single fibres as well as on their geometrical arrangement. The mechanical behaviour of fibrous materials deviates significantly from that of traditional materials because of the discontinuous nature of randomly distributed fibres. Despite their high strength, little information is available about the deformation mechanism of spun fabrics; the load bearing capacity of these materials critically affects many technological and biomedical applications. It is therefore important to know how microscopic failure processes (for example rupture of overloaded fibres) gives rise to macroscopic deformation.

The present experimental study was motivated by the recent progress in the development of fibre bundle model (FBM) which is the most fundamental model of failure^1^. This model was introduced by Pierce in 1926 to understand the strength of cotton yarns^2^. One of the first theoretical approach was introduced by Peires in 1927^3^. The planar fiber network mechanics has been studied in details by Cox HL by shear lag model^4^. The FBM models were later modified and generalised to explain a wide variety of phenomena like microscopic mechanism of fatigue, breaking of fibres in mingled mat, traffic flow, and landslides (Daniels and Coleman)^5,6^. These different phenomena are believed to have a universal feature and require a unifying model of failure formation covering a wide range of length, energy, and time scales^1–8^. Several analytical and numerical approaches have been adopted through statistical considerations. Despite experimental investigations, comparisons of the FBM with real experiments are scarcely found in the literature^9,10^.

The study aims to understand the problem of damage and fracture of disordered materials subjected to an external force.

The main purpose of the present study is to perform experiments that compare short scale damage formation with the loading curve and to provide experimental results for a possible probabilistic approach to the FBM. This model envisages a material as a set of *N* parallel fibres clamped at one end, with an external load applied at the other end. All the fibres are characterised by linear elastic behaviour with identical stiffness and have stochastic breaking thresholds. The bundle is loaded parallel to the fibre direction. The different models of fibre failure can be divided into several categories depending on the redistribution mechanism of stress released among the intact fibres. The widely accepted and most simple category is global load sharing, which assumes that the weakest fibre ruptures when the bundle is strained, and the load is equally shared by all the intact fibres. Since the Young’s modulus of the fibre bundle is proportional to the number of intact fibres that carry the load, the modulus of the fibre bundle decreases after each rupture. Consequently, the stiffness reduces, and the fabric cannot fully recover its original dimensions when the stress is terminated. According to the FBM, in a strain-controlled experiment, the average force *F(x)* at extension *x* can be expressed as:

1 $$F(x)=Nkx[1-P(x)],$$

where *N* is the number of fibres and *k* is the stiffness of individual fibres. *P(x)* denotes the cumulative probability distribution of fibre failure thresholds, and the term *N[1 - P(x)]* is the fraction of fibres that are intact at deformation x. The texture of the fibre bundle plays a decisive role in determining the form of *P(x)*, and consequently affects the shape of the loading curve. An analytical solution of Eq. 1 can be obtained if uniform distribution of fibre strength is considered. In this case *F(x)* is a smooth, symmetrical parabolic function^1^. If we assume a different probability distribution for fibre failure, numerical simulation is a possible method for solving Eq. 1 and obtaining the loading curve. It is therefore important to find the correspondence between the texture of the material and the probability distribution function. Much attention has been given recently to the application of different statistical approaches to the FBM, but any comparison of the theory with experiments is still lacking.

## Experimental

Electrospun nano- and micro-fibre networks were prepared from a synthetic polymer polysuccinimide (PSI)^11^. This polymer is the anhydrous form of poly(aspartic acid), a promising candidate for several biomedical applications like scaffold for cell proliferation and artificial extracellular matrix^12^. It is synthesised by thermal polycondensation of L-aspartic acid. PSI- dimethylformamide (DMF) solutions were filled into a glass syringe with a metal Hamilton tip and placed into a syringe pump. The positive electrode was attached to the metal tip whereas the negative electrode (ground) was attached to the collector, made of tinfoil, in front of the needle. When a DC voltage of *9 kV* was applied, the fibres were attracted to the collector. After the preparation, the fibre mat was placed in vacuum to remove remaining solvent and other impurities. The detailed procedure can be found in our previous paper^13^.

The optical micrograph was made by Hund H 500 optical microscope in magnification 4. For scanning electron microscopy image the sample were mounted on a standard sample holder by conductive adhesion graphite-pad (Plano) and examined with a Zeiss LEO 1530 (FE-SEM with Schottky-field-emission cathode; in-lens detector, SE2 detector or Back Scattered Detector) using an accelerating voltage of 2 kV. The samples were sputtered with platinum (1.3–2 nm using a Cressington HR208 sputter coater and a Cressington mtm20 thickness controller).

Unidirectional strain-controlled experiments were performed on the electrospun fibre mats. The sample was cut into rectangular specimens of 22 mm width using a razor guided by a straight edge. The distance between the two grips was 50 mm. The surface mass density of the fiber layer was 15.9 g/m^2^. The tensile tests were conducted using an Instron 5942 testing machine with a 50 N load cell. The accuracy of the load cell was ≤0.25% of the indicated force. All the stress-strain measurements were conducted at room temperature at a rate of elongation of 0.5 mm/min.

## Results and Discussion

**Figure 1:**
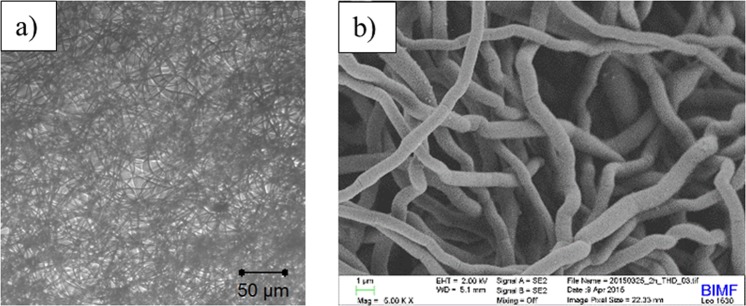
Optical microscope image (**a**) and SEM image (**b**) of a weak layer of electrospun polysuccinimide web.

Figure 1 shows the optical micrograph and the scanning electron microscope (SEM) image of the PSI web. The mats are random layered assemblies of fibres held together by non-bonded interactions, friction, and adhesion.

The individual fibres are highly entangled and the layer is isotropic. Different level of interactions can occur in the texture. Geometrical limitations as well as non-bonded interactions between the contact points of adjacent fibres restrict the movement of the fibres in the texture.

**Figure 2:**
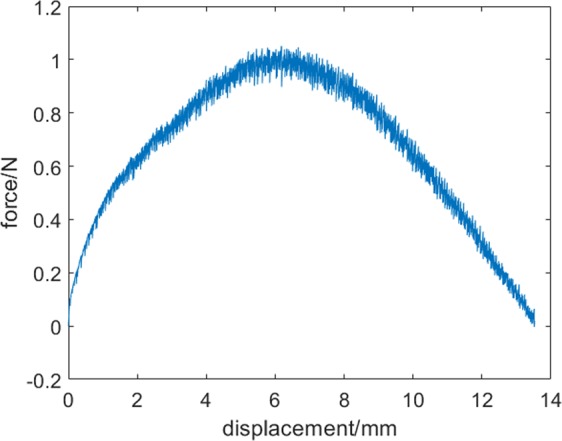
Load−displacement curve of a typical dry fibre mat.

The load−displacement curve of the sample is shown in Fig. 2. The dependence of uniaxial deformation on the load is not linear; it goes through a maximum. This type of dependence is expected on the basis of FBM under uniform distribution of fibre strength. It is noteworthy that the load−displacement curve shows quite high fluctuations in the direction of force. The amplitude of the force fluctuation was found to be much larger than the experimental accuracy.

**Figure 3:**
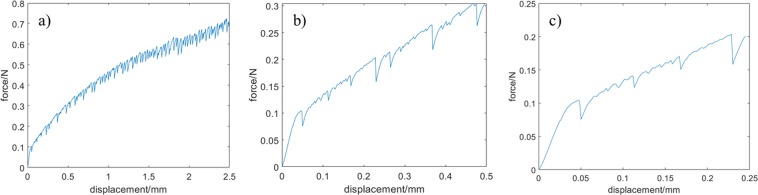
Enlarged view of the load−displacement curve of the sample shown in Fig. 2.

Detailed analysis of the shape of the loading curve was carried out by enlarging parts of the curve. Figure 3 shows the magnified plots of the load−displacement curve in the displacement range of 0–2.5 mm (Fig. 3a), 0–0.5 mm (Fig. 3b), and 0–0.25 mm (Fig. 3c). All the magnified curves exhibit saw tooth-like force−extension behaviour. This extraordinary shape indicates damage; there are abrupt drops in the force in the load−displacement curve. The force drop may be the consequence of fibre rupture or fibre slip. At this point, it is difficult to differentiate the two cases, and both of them are considered as material damage. We can conclude that, these damages become “observable” and measurable by means of our experimental technique. Thus, it is possible to quantify damage formation by analysing the measured load−displacement curve.

**Figure 4:**
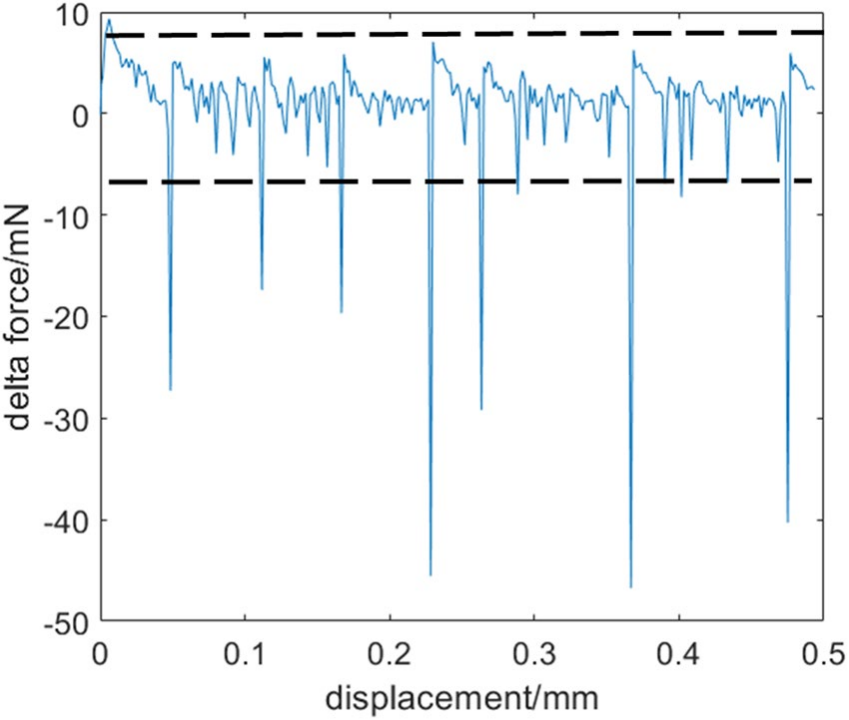
Rupture force sequences recalculated from Fig. 3b. The dotted line indicates the range of experimental error. Within this range, no force drops were taken into account.

We also investigated the statistical properties of rupture sequences in extension by studying both the position and the strength distribution of rupture force. Numerical differentiation of the loading force with respect to the displacement provides a better understanding of the dependence of rupture force on the displacement, as shown in Fig. 4. Here, we have used the load−displacement curve shown in Fig. 3b. To establish the experimental error, we analysed the *f* ≥ 0 region, where there are no force drops. Considering that the fluctuation on both sides of the force is governed by the same law, we found that the force randomly fluctuates in the range of −7.1 ≤ Δ*f*_*error*_ ≤ 7.1 mN (Fig. 4).

This interval is considered to be the experimental error. If the negative force drop exceeds −7.1 mN, then the number of such drops is accounted as damage. We also determined the magnitude of Δ*f* by taking into account Δ*f* < Δ*f*_*error*_. As evident from Fig. 4, the force drop (Δ*f*) varies from 10 to −50 mN. If *s* denotes the number of bonds damaged for a given elongation, then *s* = 7 for the enlarged case shown in Figs 3b and 4.

**Figure 5:**
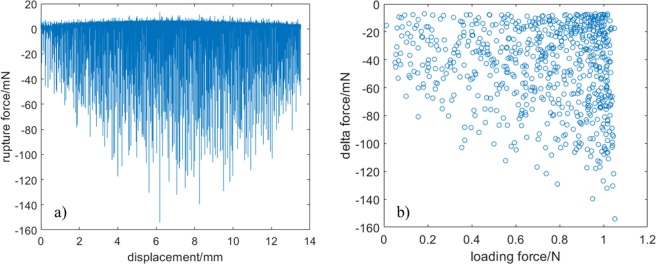
Rupture force as a function of (**a**) displacement and (**b**) loading force.

We determined the rupture force sequences for the entire deformation, and the results are shown in Fig. 5a. The (x, Δ*f*) coordinates of the loading curve after subsequent ruptures is shown in Fig. 5b. The force sequence shown in Fig. 5a is a consequence of maximum type of loading curve shown in Fig. 2. It is evident from the figures that both the appearance and the magnitude of the force drops are random. Damages occur in the fibre bundle at the very beginning of the deformation and continue until all the fibres fail. The damage evolution increases with increase in the elongation, and the range of force fluctuation increases with increase in the load. At the beginning of elongation, the force drop fluctuates between 0 and −50 mN. As the stretching increases, the magnitude of fluctuation continuously increases in the range of 0 to −150 mN. These values suggest that the strength of individual fibres is different; each fibre has a different brittle response until it breaks. When the bundle is strained, the weakest fibre begins to rupture, followed by other fibres in the order of increasing strength. Thus, a wide spectrum of the elastic constant of fibres has to be taken into account. This spectrum enable us to determine the number of failures as a function of the displacement as well as the magnitude of rupture force as a function of the applied external force. For example, in this sample, the force drops exceeding −7 mN was found to be s = 683.

**Figure 6:**
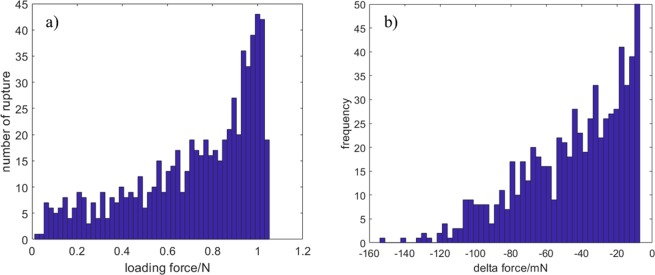
Frequency histogram of (**a**) number of ruptures and (**b**) magnitude of rupture force.

To visualise the general characteristics of numerical data and to identify the different mechanical behaviour, we summarised our experiment results by histograms. Figure 6a shows the number of ruptures as a function of the loading force, and Fig. 6b shows the frequency of distribution of the magnitude of rupture force. The bin width is 50 in both the cases. These histograms represent an approximate probability density function that can be used to determine a possible probabilistic approach to the FBM.

### Summary of the Main Results

The unidirectional deformation of weak, planar, randomly oriented electrospun fibre networks was studied. Strain-controlled loading was monitored in the presence of damage formation. With increase in the elongation, the weakest fibre begins to rupture, followed by other fibres in the order of increasing strength. We analysed randomly appearing damage formation by measuring subsequent force drops as a consequence of rupture of fibres. We quantified the complex phenomena by determining the number and the magnitude of the force drops. We found that the weak fibre network exhibits a complex, nonlinear stress-strain behaviour with a maximum parabolic type loading curve. The mechanical behaviour of this random structure is discussed in the framework of the FBM; this will help in analysing the damage and fracture of disordered materials subjected to an external force. The frequency of rupture as well as the frequency of rupture force is represented by histograms, which helps in development of possible probabilistic FBM models and identification of unusual behaviour. Our experimental method has the potential for the development of a statistical model based on direct comparison of experimental results with model simulations and can contribute to the design of high-performance fibre mats. However, further experimental studies on systematic variation of fibre texture combined with numerical simulations and statistical physics models on damage evolution are required.

## Conclusion

An experimental technique has been proposed to study the effect of material damage on the stress–strain behaviour of weak, planar electrospun fibre networks. The results of strain controlled mechanical experiments showed saw tooth-like force−extension behaviour at short scale and maximum parabolic type loading curve dependence at large scale. This complex, nonlinear stress-strain behaviour is a consequence of rupture of fibres during extension. The number of force drops following each fibre rupture as well as their magnitude were measured and analysed. The approximate probability density function of fibre rupture, derived from mechanical experiments, was represented using a histogram. This experimental technique has significant potential for the characterisation of fibre texture and suggests a possible probabilistic approach to the FBM.
